# The Health Equity Leadership Institute (HELI): Developing workforce capacity for health disparities research

**DOI:** 10.1017/cts.2017.6

**Published:** 2017-06-19

**Authors:** James Butler, Craig S. Fryer, Earlise Ward, Katelyn Westaby, Alexandra Adams, Sarah L. Esmond, Mary A. Garza, Janice A. Hogle, Linda M. Scholl, Sandra C. Quinn, Stephen B. Thomas, Christine A. Sorkness

**Affiliations:** 1 Department of Behavioral and Community Health, School of Public Health, Maryland Center for Health Equity, University of Maryland, College Park, MD, USA; 2 School of Nursing, Collaborative Center for Health Equity, University of Wisconsin, Madison, WI, USA; 3 Wisconsin Partnership Program, School of Medicine and Public Health, University of Wisconsin, Madison, WI, USA; 4 Department of Family Medicine, School of Medicine and Public Health, University of Wisconsin, Madison, WI, USA; 5 Institute for Clinical and Translational Research, Collaborative Center for Health Equity, School of Medicine and Public Health, University of Wisconsin, Madison, WI, USA; 6 Institute for Clinical and Translational Research, School of Medicine and Public Health, University of Wisconsin, Madison, WI, USA; 7 Department of Family Science, Maryland Center for Health Equity, School of Public Health, University of Maryland, College Park, MD, USA; 8 Health Services Administration, Maryland Center for Health Equity, School of Public Health, University of Maryland, College Park, MD, USA; 9 Institute for Clinical and Translational Research, Collaborative Center for Health Equity, University of Wisconsin, Madison, WI, USA

**Keywords:** Health equity research, career development, health disparities, diverse research workforce

## Abstract

**Introduction:**

Efforts to address health disparities and achieve health equity are critically dependent on the development of a diverse research workforce. However, many researchers from underrepresented backgrounds face challenges in advancing their careers, securing independent funding, and finding the mentorship needed to expand their research.

**Methods:**

Faculty from the University of Maryland at College Park and the University of Wisconsin-Madison developed and evaluated an intensive week-long research and career-development institute—the Health Equity Leadership Institute (HELI)—with the goal of increasing the number of underrepresented scholars who can sustain their ongoing commitment to health equity research.

**Results:**

In 2010-2016, HELI brought 145 diverse scholars (78% from an underrepresented background; 81% female) together to engage with each other and learn from supportive faculty. Overall, scholar feedback was highly positive on all survey items, with average agreement ratings of 4.45-4.84 based on a 5-point Likert scale. Eighty-five percent of scholars remain in academic positions. In the first three cohorts, 73% of HELI participants have been promoted and 23% have secured independent federal funding.

**Conclusions:**

HELI includes an evidence-based curriculum to develop a diverse workforce for health equity research. For those institutions interested in implementing such an institute to develop and support underrepresented early stage investigators, a resource toolbox is provided.

## Introduction

One of the overarching goals of the US Department of Health and Human Services’ *Healthy People 2020* plan is to achieve health equity, eliminate disparities, and improve the health of all groups. As many have noted, a critical component in efforts to reduce health disparities and achieve health equity is the development of a diverse research workforce [1–3]. Expanding the racial/ethnic, socioeconomic, and sociocultural backgrounds of the research community can extend the scope of questions investigated and bring innovative methodologies to the biomedical sciences [4]. Currently, a number of groups are underrepresented in the biomedical research workforce, including African Americans, Latinos, Native Americans and Alaska Natives, Pacific Islanders, and multiracial scientists. Not only do such researchers bring their commitment and life experiences to eliminating health disparities, but they also serve as role models and mentors for young scholars from underrepresented groups [5–7]. Despite clear evidence that scholars from underrepresented backgrounds are committed to health disparities research and service to their communities[5, 6], as researchers, they remain substantially underrepresented, particularly in academic and biomedical institutions.

Multiple studies have identified barriers faced by underrepresented groups for advancement and successful funding. For example, underrepresented researchers are less likely to be promoted than their White colleagues [8, 9], and to obtain National Institutes of Health (NIH) funding, even after accounting for differences in training, experience, and productivity [10]. Other barriers include the lack of institutional support and poor or nonexistent mentoring required for successful career advancement [3, 4]; overt discrimination and unconscious bias [11, 12]; loneliness and isolation within academic settings [4, 5]; disregard for their research interests; and expectations that they will lead university “diversity” efforts or serve as experts in all issues related to race or ethnicity [6, 11, 13]. In the face of such challenges, some scholars from underrepresented groups choose to leave academic institutions. Still others decide not to “rock the boat” in an effort to advance within the institution, while many express a desire for guidance from culturally responsive mentors to navigate and succeed in their paths [11, 13, 14].

## Methods

### Development of Health Equity Leadership Institute (HELI)

In order to address the compelling need among health equity researchers for career development and mentorship, the University of Maryland’s Center for Health Equity (M-CHE) and the University of Wisconsin-Madison’s Collaborative Center for Health Equity (CCHE) developed an intensive week-long research and career development institute with the broad goal of increasing the number of investigators—particularly those from underrepresented or disadvantaged backgrounds—engaged in health disparities and health equity research who successfully compete for tenure track academic positions and independent federal funding. Grounded in the M-CHE faculty’s previous Summer Research Career Development Institute [15], HELI was purposely designed as a structured means for bringing together investigators from various disciplines. Based on our experience and to distinguish our work from other programs, we knew that didactic lectures and sessions with limited interpersonal interaction would be inadequate. As such, HELI was designed to be a supportive and engaging environment in which participants could bring their full identities—professional and personal—to share experiences of marginalization and to jointly strategize methods for overcoming career barriers at their home institutions. This article describes key components of the institute and its programming, with special emphasis on those transformative elements that have contributed to its success. We also provide descriptive data about the scholars, evaluation data related to their HELI experience, and follow-up data on the scholars’ career paths since participating in the Institute.

### Application and Selection Process

The HELI Call for Applications is distributed widely in the early spring, with outreach efforts focused on inviting applicants from both research-intensive and minority-serving institutions. The call specifically invites applications from early career researchers with a demonstrated commitment to eliminating health disparities through a record of work in this field. Applicants submit an online application, their curriculum vitae, a letter of support from a research mentor or department head, and a personal statement about their research interest and experiences. Faculty leads make the final selection with the goal of identifying a diverse cohort of scholars representing different disciplines including public health, medicine, clinical sciences, behavioral health, and the social sciences. Researchers from University of Wisconsin-Madison (UW-Madison) and the University of Maryland are deliberately encouraged to apply to advance their careers at our parent institutions. Prior to HELI, scholars and presenters receive biographical information, including headshots, about all attendees. This practice helps begin the community-building process and jumpstart substantive scholar interaction, engagement, and networking once they are on site.

### The HELI Scholars

A total of 145 researchers were selected as HELI scholars for the 2010–2016 cohorts, ranging from 22–26 participants per year. In 2014, instead of selecting a new cohort, the faculty directors invited all the HELI scholars from previous years to participate in an alumni HELI with programming specifically tailored for that group. Table 1 provides demographic and background data on all scholars to date.

**Table 1:** Health Equity Leadership Institute (HELI) scholars (2010–2016) (n=145)

	n	%
Gender		
Female	117	81
Male	28	19
Age		
≤30	11	8
31–40	97	67
>40	37	26
Race/ethnicity (multiple selections possible)		
Black or African American	70	43
White	36	22
Asian	21	13
Hispanic/Latino	18	11
American Indian or Alaska Native	8	5
Other	9	6
From disadvantaged background*	50	34
Type of institution		
Doctoral universities—research intensive	101	70
Four-year medical schools and centers	21	14
Master’s colleges/universities	10	7
Baccalaureate colleges	3	2
Other†	10	7
Historically Black colleges and universities	6	4
Hispanic-serving institutions	3	2
Minority-serving institutions	24	17
HELI partnership university‡	37	26
Clinical and Translational Science Award awarded institution§	66	46
Title		
Postdoctoral fellow/scholar	42	29
Instructor/lecturer	4	3
Assistant professor	77	53
Associate scientist	4	3
Associate professor	4	3
Other	14	10
Highest degree attained		
Ph.D.	110	76
M.D.	12	8
M.D./Ph.D.	4	3
Dr.P.H.	14	10
Ed.D.	2	1
Master’s	3	2
Communities of interest (multiple selections possible)		
Black or African American	72	50
Hispanic or Latino	39	27
Asian	18	12
American Indian or Alaska Native	14	10
White	14	10
Other‖	22	15
Primary research category (multiple selections possible)		
Community-based participatory research	54	37
Population health/epidemiology	26	18
Public health	23	16
Behavioral health	20	14
Health services	10	7
Clinical	9	6
Laboratory-based	4	3
Other/not specified	9	6
Grant funding at time of HELI participation (multiple selections possible)		
Departmental funds	35	24
Federal¶	30	21
Institutional pilot funds	18	12
Foundation/non-profit	15	10
Miscellaneous local funds	4	3

* Disadvantaged background was self-identified.

† For example: National Institutes of Health (NIH), health departments, private/non-profit research centers.

‡ University of Wisconsin-Madison and University of Maryland, College Park.

§ Institutions with a Clinical and Translational Science Award from the NIH.

‖ Economically disadvantaged communities, Afro-Caribbean, Appalachian, Burmese Refugee, East African immigrants, Middle-eastern/Arab, incarcerated individuals, refugees with disabilities, sexual minorities, veterans/military populations, and women.

¶ Federal funding includes Career Development Awards.

### The HELI Curriculum

HELI is held over a 5-day period on the campus of the University of Wisconsin-Madison. Faculty from the National Institute of Minority Health and Health Disparities (NIMHD) Centers of Excellence at Wisconsin and Maryland serve as core faculty facilitators and key resources for the HELI scholars. Additional instruction is provided by a former research scientist and NIH program officer in the Division of Cancer Control and Population Sciences at the National Cancer Institute. Faculty and deans from UW-Madison’s School of Medicine and Public Health and Institute for Clinical and Translational Research (ICTR) also highlight the university’s research environment.

Sessions (between 1 and 2 hours each) cover a variety of topics: translational research and health equity, integration of personal and professional lives, career development, funding, mentoring, and leadership. Sessions are designed to maximize participant interaction and discussion. All scholars attend all sessions; however, scholars may schedule individual meetings with the core faculty facilitators to discuss their specific career and research trajectories and with the NIH expert to discuss their research and potential funding mechanisms. Although specific sessions have varied somewhat from year to year based on feedback from the scholars, the core HELI curriculum outlined in Table 2 has remained stable. As can be noted, the HELI curriculum includes many sessions that one would expect to see in any institute focused on career development for junior health equity investigators. What may not be so evident is the attention devoted in HELI to promoting the integration of the personal and the professional dimensions. Throughout the institute, scholars are encouraged to reflect on and share how their lived experiences, both within and beyond their academic institution, intersect with their health equity research foci and community interests. This practice is based on the idea of “centering in the margins”—a key concept in critical race theory that shifts “a discourse’s starting point from a majority group’s perspective…to that of the marginalized group or groups… By grounding themselves in the experiences and perspectives of the minority communities from which they largely come, critical race theorists integrate critical analyses of their lived experiences and disciplinary conventions to advance knowledge on inequities” [26]. In designing HELI, faculty have utilized a “centering in the margins” approach by attempting to create a safe environment in which participants can openly engage with and support one another, discuss their commitment to research with minority communities as well as their feelings of isolation and marginalization within the academy, and strategize methods for overcoming these significant career barriers. Accordingly, the HELI faculty share their own range of experiences as health equity researchers, validate the scholars’ feelings, and provide their insights into how scholars can remain committed to health equity research while still advancing their careers.

**Table 2:** The Health Equity Leadership Institute (HELI) curriculum

Translational research and health equity
1. Building trust between minorities and researchers: presentation on how the social and historical context affects the research interaction between potential participants and researchers [16–19]
2. Translational research across the color line: the conceptual framework of 3 generations of health disparities research is examined to understand (a) data trends, (b) factors driving disparities, and (c) solutions for closing the gap. A fourth generation of research is proposed, specifically research grounded in public health critical race praxis, and interventions to address race, racism, structural inequalities, and the researcher’s own biases [20]
3. Connecting with communities: multiple panel presentations throughout the 5-day HELI institute on a wide range of community-based health equity research. This includes a full-day trip to Milwaukee, WI to visit UW-Madison’s community-engaged research partnerships. The trip affords not only a chance to visit and learn from partner communities, but also to engage in additional social connections among scholars and faculty
Personal/professional development
4. Research scholar presentations: presentations by each scholar structured as a 4-slide PowerPoint addressing: (1) who I am, (2) what I do, (3) why it matters to health equity, and (4) what I need. Several presentations are assembled as a session, and the sessions are integrated into the programming in the first 2 days of the institute in order to facilitate community building among scholars
5. Scientific autobiographies: core faculty facilitators model a reflection on factors that have shaped their identities as underrepresented investigators. Scholars then share their own reflections on connections between their career passion and their life experiences
6. Work-life integration and leadership: session highlights personal growth and leadership, personal values, health and wellness, and the concept of life/work integration
Career development and funding strategies
7. Preparing for tenure review: advice from faculty on advancing through the promotion and tenure process, understanding an institution’s tenure guidelines, utilizing mentors, structuring and promoting a program of research, and highlighting community-engaged research partnerships
8. Acquiring and transitioning from a career development award: panel presentation on optimizing the chances of obtaining a career development award; making the most of one’s time on a career development award; and successfully transitioning off career development award funding
9. NIH grant writing, grantsmanship, and grant submission: overview of NIH grant solicitations and mechanisms, including those for health equity research, with an emphasis on the mentored career development awards, fellowships, diversity supplements, and research program grants
10. Mock NIH study sections: 2 HELI scholars are chosen to have their grant proposals reviewed in an open setting. Reviewers follow the standard study section format with HELI scholars as observers. At the end of the session, the chosen scholars reflect on the feedback
Mentoring
11. Tools and strategies for getting the most from mentor-mentee relationships: participants will reflect on the kind of mentoring they need now and will need in future stages of their careers, consider issues to discuss when establishing positive and open mentor-mentee relationships (“mentor” vs. “tormentor”), identify and prioritize the roles that mentors can play in their career, and discuss important factors to consider in mentoring relationships that are built around health disparities/health equity focused research [21–23]
12. Cultural awareness in mentoring relationships: this session addresses diversity issues in science with a focus on fixing environments and systems through a series of small steps that individuals take in their mentoring relationships. HELI scholars are asked to reflect on and discuss their own racial and ethnic identity and work collaboratively to explore strategies to address race and ethnicity in their mentoring relationships, both as a mentor and as a mentee [24, 25]
13. Balancing perfection and productivity: in this session, panelists discuss common pitfalls that junior faculty members may face related to finding the right balance between teaching, service and research; for example: acquiring a rudimentary understanding of an academic home; poor mentoring experiences; uneven collegial support; and overlooking the importance of discipline and vigilance in the pursuit of grant support, publishing, and teaching excellence
Leadership
14. Scientific management skills 101: a panel of junior and senior investigators discusses skills necessary for managing a grant including hiring and overseeing personnel, purchasing, subcontracts and managing the grant budget, preparing progress reports, and ensuring protected time to meet the specific aims
15. Leadership competencies: discussion of core principles of leadership and case studies of common leadership challenges

NIH, National Institutes of Health.

This approach is utilized early in the HELI application process, and reinforced through the Research Scholar Presentations (Table 2, no. 4) in which each scholar presents to the group about who they are, what they do, why it matters to health equity, and what they need. After each presentation, the group is given an opportunity to ask more questions, provide resources, and make connections to their own or others’ work and experiences. These presentations are held early in the institute to facilitate rapport and trustworthiness among the cohort and faculty, as well as the development of professional and personal connections—connections that often endure far beyond the 5-day institute.

Another key session that highlights HELI’s “centering the margins” approach is the Scientific Autobiographies session (Table 2, no. 5) in which scholars are invited—after hearing core faculty facilitators model the practice—to reflect on factors that have shaped their identities as health equity investigators. The term “scientific autobiography” is not new; it has been used by teachers to capture their personal experience with science in autobiographical essays [27], by bench scientists to describe the evolution of their scientific work [28], and by participants in the Summer Research Career Development Institute to appreciate models of academic success in minority health disparities [15]. At HELI, the format is designed to help scholars articulate their career passion tied directly to their life experiences. Over the course of 6 years, the Scientific Autobiographies session has become a highly popular session as it has provided dedicated time and space for scholars to directly engage the “whole-self” and not just their “professional self” by sharing personal experiences, learning, and resilience [29].

In addition to these sessions, scholars are encouraged to participate actively in each day’s programming by raising questions, making connections with their own experiences, supporting and validating each other’s work, and reflecting on the intersection between their personal and the professional lives. Each year, this has resulted in the creation of a co-learning environment and the development of a tangible and often powerful sense of community. Scholars have typically extended each day’s programming by self-organizing evening social gatherings in which the conversations are continued.

### Staying Connected with the HELI Community

Following HELI, the CCHE team maintains connectivity with and among scholars using a variety of mechanisms: a HELI WordPress blog, a Facebook (FB) page, and targeted emails. The FB page was developed as a virtual setting for connecting HELI staff, faculty, and scholars. Posts on the FB page include national health equity and minority health research job opportunities, articles on health disparities authored by the scholars and others, and announcements of alumni promotions and successes. Some posts from scholars are inquiries to the HELI community for guidance about a particular topic (mentor selection, challenging work situations), technical assistance, or research expertise unavailable at a scholar’s institution. The HELI WordPress blog (uwheli.com) was established to raise the institute’s visibility and to provide online resources for prospective scholars to access application materials, HELI agendas, and alumni profiles. In addition, CCHE staff and faculty of UW-Madison and M-CHE send HELI scholars targeted emails containing information about relevant health equity research trainings, webinars, and lectures, as well as employment and career development opportunities. Furthermore, scholars have secured letters from HELI faculty for promotion and tenure reviews. HELI scholars often connect with each other or attend CCHE-sponsored annual reunions at national research conferences (eg, American Public Health Association, NIMHD Summit on the Science of Eliminating Health Disparities), or via participation in National Research Mentoring Network (NRMN) programs.

### Program Evaluation Framework

From the beginning of the institute, HELI staff and faculty have collaborated with the UW ICTR Evaluation Office to assess the institute. Using a mixed methods approach, the evaluation aims to collect scholars’ feedback about the sessions and the overall institute to improve the programming and to track the scholars’ careers and research following HELI participation. Evaluation data are gathered in multiple ways. Prior to HELI, scholars are sent a survey, asking them about their level of satisfaction with their current academic position, particularly related to mentoring and integration into their departments. The survey also asks them what they hope to gain from HELI participation. During the institute, daily electronic evaluations are sent to the scholars at the close of each day to collect immediate feedback about the presentations. HELI facilitators carefully review and discuss scholars’ feedback about the institute; iterative changes to sessions and to the overall flow of the institute have been made based on this feedback. The final day’s survey includes questions about the overall HELI experience. Also on the last day, a final feedback session is held. Scholars sit in a circle with the faculty, passing a “talking stick” (a Native American tradition symbolizing the right to speak) to comment on their HELI experience. Many scholars take the opportunity to describe what they learned at HELI and what it meant to them professionally and sometimes personally. Finally, an annual follow-up survey is sent to scholars to gather information about their career trajectories, mentor experiences, changes to their institutional affiliation, success in achieving grant funding, and publications. Scholars are also asked about the impact of HELI on their career development and commitment to health equity research. Additional data on academic promotion, career persistence, and funded grants are obtained from public Web sites (eg, NIH RePORTER). All data are collected as program evaluation and thus not classified as human subjects research.

## Results

A comprehensive presentation of evaluation data from HELI is beyond the scope of this manuscript and will be shared in a future publication [30]. Nevertheless, we highlight (1) overall feedback from the scholars garnered in the final surveys and feedback sessions over the past 2 years as HELI has reached maturity, and (2) follow-up data on the scholars that have participated in HELI since 2010.

Table 3 shows evaluation results from the 2 most recent years of overall feedback by the scholars about their HELI experience. Feedback was highly positive on all survey items, with average agreement ratings between 4.45 and 4.84 based on a 5-point Likert scale. Scholars consistently report that HELI provided useful information about the social, economic, and cultural determinants of health and research approaches that could be used to better understand and address health disparities. They also consistently indicate that they received useful career and research guidance through the institute and that HELI promoted a safe environment for sensitive discussions related to isolation and discrimination.

**Table 3:** Evaluation results from 2015 and 2016 scholars’ Overall Feedback Survey (n=43–47 responses)

Survey item	Mean*	SD
HELI allowed scholars to freely discuss feelings of isolation	4.78	0.51
HELI allowed scholars to freely discuss feelings of discrimination	4.74	0.59
HELI created a safe environment for sharing	4.72	0.84
HELI adequately addressed the social, economic, and cultural determinants of health that contribute to disparities	4.52	0.54
I received useful career and research guidance to develop research programs focused on health disparities and health equity	4.84	0.37
I can identify determinants of health that contribute to the health disparity gaps	4.58	0.72
I understand evidence-based research approaches that are applicable for use	4.45	0.62

HELI, Health Equity Leadership Institute.

* Based on a 5-point Likert scale: 5=strongly agree, 4=agree, 3=neither agree nor disagree, 2=disagree, 1=strongly disagree.

Open-ended responses from the surveys and group feedback sessions provide rich data on scholars’ views of their HELI experience. The responses below were chosen from a large repository of scholars’ comments because they reflect sentiments that HELI facilitators have heard repeatedly over the years.

*“Attending HELI made a significant impact on my development as a health equity and childhood obesity researcher. [The sessions] helped me understand how to map out my 5- and 10-year plans, identify and begin relationships with new institutional mentors, and begin preparing for my promotion and tenure review process at the start of my new position. Of equal importance, I now have a network of peers who have an appreciation of and dedication to health equity research; this not only includes HELI scholars from my own cohort, but also scholars from other cohorts with whom I have connected in other settings.”*

*“HELI connected me to other health equity scholars and experts in the field, which validated my work and provided opportunities for expanding my own work. The topics on grant writing, tenure, and methods were particularly helpful. However, the most powerful aspect of HELI was the emotional connection it facilitated among a group of individuals who are doing amazing work but who feel isolated or unsupported in their work due to institutional or societal barriers.”*

*“The thing I would like to see you keep the most is the sense of safe space and the ability to talk about race issues and some of the experiences we’ve had that we can’t really talk about in our institutions. The isolation that some of us feel and deal with and bring here and actually have like minds and the networking that happens here is, for me, one of the most important things that I got out of this. You can learn how to write a grant somewhere else, but the safe space that’s here, the ability to share and have people that understand what you’re saying and not look at you like you’re odd. To be able to help you think through some of those situations when you’re having problems or difficulties or you’re facing that situation that feels like racism, you can bounce it off someone else and say, ‘Now what am I supposed to do with this and how do I fix this?’ That’s the most valuable thing I got here.”*

*“For the first 3 years of my tenure track position, I felt very incompetent. So I went to every training under the sun… I came to HELI 2010, the inaugural group, and…at the end…I said, ‘I finally feel like I am competent, like I can do this work.’ It was all of the discussions that we were having and just feeling validated. Other folks were saying the same things and experiencing the same things. When I left I finally felt competent and I just sailed on from there.”*

*“HELI was an important experience for me for [several] reasons: one, it helped me form contacts with folks I still see at conferences; two, it provided insight (and commiseration) for what I and other early-career folks are going through; three, it encouraged me to retain focus on issues of health equity and community research. And four, I received helpful feedback on a grant proposal.”*

*“What distinguishes [HELI] is the intentional and deliberate nature of intentional reflection. The intentional pre-survey and pre-work we had to do in advance. We hung out every night. The reflective nature is the secret sauce. You take us out of our silos where we’re all machines and let us just be. I said I needed to recharge when I came here. This is a space of collective strength and support.”*

**Fig. 1:**
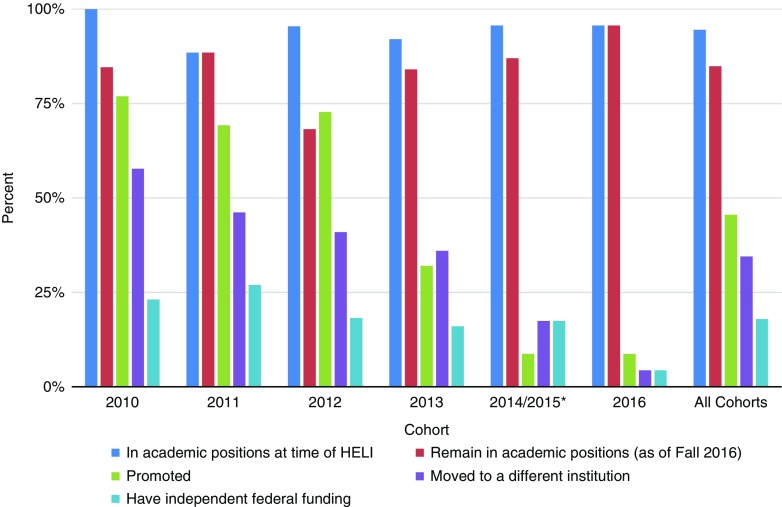
Percent of Health Equity Leadership Institute (HELI) scholars by cohort with various academic career indicators. *In 2014, there were 3 new HELI scholars; the other participants in that year were alumni from previous years. The 2014/2015 cohort thus combines data from the 3 new scholars in 2014 with the 20 scholars in 2015.

Finally, Fig. 1 provides follow-up information about the scholars’ career trajectories by cohort. The outcomes that we have chosen to track represent those that are most commonly and conservatively associated with successful career development in the biomedical sciences: retention in the workforce, career promotions, and the attainment of independent, federal funding. These data do not rely on self-report from the scholars, but rather are independently verified via public sources.

Overall, 85% of scholars who were in academic positions during HELI participation remain in academic positions as of December 2016. Seventy-three percent of HELI scholars from the first 3 cohorts (2010, 2011, and 2012) have been promoted; 23% have secured independent federal funding. Notably, this latter measure does not reflect the total percent of scholars who have secured independent non-federal funding. Anecdotally, we know that many HELI scholars have diversified research funding portfolios drawn from non-federal sources (eg, foundations, private research centers, institutional initiatives).

## Discussion

Although a 5-day institute such as HELI is unlikely, in itself, to lead to career success and satisfaction for junior investigators in the field of health equity, it can play an important role to welcome, engage, mentor, and promote the careers of junior investigators conducting health equity research with marginalized communities. As we have noted at HELI, the barriers mentioned at the beginning of this article continue to impact the lives of these junior investigators. By creating an open and inclusive environment, HELI attempts to provide scholars with social support they need to address these barriers and challenges. HELI not only engages scholars in robust discussions about health equity research with underrepresented communities and mechanisms that can fund their research, but also provides career guidance, leadership development, mentoring, strategies for work-life integration, and a close community of scholars that serve as mutual sources of support, validation, and resilience. Indeed, what makes HELI distinctive is the creation of a trusting, open atmosphere in which racism, painful experiences and fears, can be shared, and young scholars feel less alone in their academic journey. Scholars have rated the institute highly and have provided extensive narrative comments about the value of their HELI experience. We remain in contact with most of the HELI scholars and have seen many advance their careers, get promoted, and secure research funding. HELI alumni are returning as institute faculty and participating as NRMN Master Facilitators and mentors. The NRMN Web site can be accessed through the URL: https://nrmnet.net/

Given the crucial importance of health equity research in improving overall human health, academic medical and public health institutions can play a key role in supporting junior investigators in this field, particularly those from underrepresented communities themselves. By developing a range of strategies and exemplary practices, institutions can promote greater inclusion of underrepresented scholars and communities, address biases and discrimination that stifle investigators’ careers, and provide critical support and culturally aware mentoring for investigators whose research will contribute to the reduction of health disparities. We have described HELI in this manuscript in order to share exemplary practices that contribute to this effort and to the overall development of a diverse health disparities workforce. In an effort to encourage other institutions to host their own version of HELI, we are sharing tools that can advise and guide implementation via the HELI Resource Toolbox (https://uwheli.com/heli-resource-toolbox/). The toolbox contents include: a sample HELI application, a sample pre-event survey, 5 years of past HELI programs, daily evaluation survey samples, and a sustainability discussion.

## References

[1] Koh HK, Graham G, Glied SA. Reducing racial and ethnic disparities: the action plan from the department of health and human services. Health affairs (Project Hope) 2011; 30: 1822–1829. 21976322 10.1377/hlthaff.2011.0673

[2] National Institute of Health. *Working Group on diversity in the biomedical research workforce* [Internet], 2011 [cited Jun 27, 2014]. (http://acd.od.nih.gov/dbr.htm).

[3] Committee to Review the Clinical and Translational Science Awards Program at the National Center for Advancing Translational Sciences, Board on Health Sciences Policy, Institute of Medicine. The National Academies Collection: Reports funded by National Institutes of Health In: Leshner AI, Terry SF, Schultz AM, Liverman CT, eds. The CTSA Program at NIH: Opportunities for Advancing Clinical and Translational Research. Washington, DC: National Academies Press (US) National Academy of Sciences, 2013: 1, 179. 24199260

[4] Leggon CB. Women in science: racial and ethnic differences and the differences they make. The Journal of Technology Transfer 2006; 31: 325–333.

[5] Shavers VL, et al Barriers to racial/ethnic minority application and competition for NIH research funding. Journal of the National Medical Association 2005; 97: 1063–1077. 16173321 PMC2575989

[6] Viets VL, et al Reducing health disparities through a culturally centered mentorship program for minority faculty: the Southwest Addictions Research Group (SARG) experience. Academic Medicine: Journal of the Association of American Medical Colleges 2009; 84: 1118–1126. 19638783 10.1097/ACM.0b013e3181ad1cb1 PMC3674962

[7] Pololi LH, et al The experience of minority faculty who are underrepresented in medicine, at 26 representative U.S. Medical Schools. Academic Medicine: Journal of the Association of American Medical Colleges 2013; 88: 1308–1314. 23887015 10.1097/ACM.0b013e31829eefff

[8] Beech BM, et al Mentoring programs for underrepresented minority faculty in academic medical centers: a systematic review of the literature. Academic Medicine: Journal of the Association of American Medical Colleges 2013; 88: 541–549. 23425989 10.1097/ACM.0b013e31828589e3 PMC3835658

[9] Yu PT, et al Minorities struggle to advance in academic medicine: a 12-y review of diversity at the highest levels of America’s teaching institutions. The Journal of Surgical Research 2013; 182: 212–218. 23582226 10.1016/j.jss.2012.06.049

[10] Ginther DK, et al Race, ethnicity, and NIH research awards. Science (New York, N.Y.) 2011; 333: 1015–1019. 10.1126/science.1196783 PMC3412416 21852498

[11] Peterson NB, et al Faculty self-reported experience with racial and ethnic discrimination in academic medicine. Journal of General Internal Medicine 2004; 19: 259–265. 15009781 10.1111/j.1525-1497.2004.20409.x PMC1492150

[12] Price EG, et al The role of cultural diversity climate in recruitment, promotion, and retention of faculty in academic medicine. Journal of General Internal Medicine 2005; 20: 565–571. 16050848 10.1111/j.1525-1497.2005.0127.x PMC1490155

[13] Mahoney MR, et al Minority faculty voices on diversity in academic medicine: perspectives from one school. Academic Medicine: Journal of the Association of American Medical Colleges 2008; 83: 781–786. 18667896 10.1097/ACM.0b013e31817ec002 PMC2868964

[14] Cropsey KL, et al Why do faculty leave? Reasons for attrition of women and minority faculty from a medical school: four-year results. Journal of Women’s Health (2002) 2008; 17: 1111–1118. 10.1089/jwh.2007.0582 18657042

[15] Berget RJ, et al A plan to facilitate the early career development of minority scholars in the health sciences. Social Work in Public Health 2010; 25: 572–590. 21058215 10.1080/19371911003748174 PMC3016049

[16] Quinn SC, Kass NE, Thomas SB. Building trust for engagement of minorities in human subjects research: is the glass half full, half empty, or the wrong size? American Journal of Public Health 2013; 103: 2119–2121. 24134371 10.2105/AJPH.2013.301685 PMC3966693

[17] Passmore SR, et al Building a “deep fund of good will”: reframing research engagement. Journal of Health Care for the Poor and Underserved 2016; 27: 722–740. 27180705 10.1353/hpu.2016.0070 PMC5502676

[18] Fryer CS, et al The symbolic value and limitations of racial concordance in minority research engagement. Qualitative Health Research 2016; 26: 830–841. 25769299 10.1177/1049732315575708 PMC4658313

[19] Quinn SC, et al Building trust between researchers and minorities [Internet]. The University of Maryland Center for Health Equity, 2013 [cited Mar 15, 2017]. (http://www.buildingtrustumd.org/).

[20] Thomas SB, et al Toward a fourth generation of disparities research to achieve health equity. Annual Review of Public Health 2011; 32: 399–416. 10.1146/annurev-publhealth-031210-101136 PMC3419584 21219164

[21] Pfund C, et al Building national capacity for research mentor training: an evidence-based approach to training the trainers. CBE Life Sciences Education 2015; 14: ar24. 26033872 10.1187/cbe.14-10-0184 PMC4477740

[22] Pfund C, et al Training mentors of clinical and translational research scholars: a randomized controlled trial. Academic Medicine: Journal of the Association of American Medical Colleges 2014; 89: 774–782. 24667509 10.1097/ACM.0000000000000218 PMC4121731

[23] Sorkness CA, et al Research mentor training: initiatives of the University of Wisconsin Institute for Clinical and Translational Research. Clinical and Translational Science 2013; 6: 256–258. 23919359 10.1111/cts.12085 PMC3979849

[24] Pfund C, et al Defining attributes and metrics of effective research mentoring relationships. AIDS and Behavior 2016; **Suppl 2**: 238–248. 10.1007/s10461-016-1384-z PMC4995122 27062425

[25] McGee R. Biomedical workforce diversity: the context for mentoring to develop talents and foster success within the “pipeline”. AIDS and Behavior 2016; 20(Suppl. 2): 231–237. 27424004 10.1007/s10461-016-1486-7 PMC4995113

[26] Ford CL, Airhihenbuwa CO. The public health critical race methodology: praxis for antiracism research. Social Science & Medicine (1982) 2010; 71: 1390–1398. 20822840 10.1016/j.socscimed.2010.07.030

[27] Koch J. The science autobiography. Science & Children 1990; 28: 42–43.

[28] Wali KC. A Scientific Autobiography S Chandrasekhar. Singapore: World Scientific Publishing Company, 2011.

[29] Shamir B, Eilam G. “What’s your story?” A life-stories approach to authentic leadership development. The Leadership Quarterly 2005; 16: 395–417.

[30] Adams A, *et al*. Assessing and communicating the value of biomedical research: results from a pilot. *Academic Medicine: Journal of the Association of American Medical Colleges.* In Press, doi:10.1097/ACM.0000000000001769. PMC5617770 28640028

